# Portal Vein Thrombosis Associated With Fusobacterium nucleatum Bacteremia: A Rare Abdominal Variant of Lemierre’s Syndrome

**DOI:** 10.7759/cureus.27918

**Published:** 2022-08-12

**Authors:** Mahsa Mohammadian, Payal Rath, Anthony Dikhtyar, Shruti Jesani, Ramez Alyacoub

**Affiliations:** 1 Internal Medicine, Rutgers Health/Trinitas Regional Medical Center, Elizabeth, USA; 2 Internal Medicine, St. George's University School of Medicine, West Indies, GRD

**Keywords:** pylephlebitis, portal vein thrombosis, fusobacterium nucleatum, fusobacterium necrophorum, atypical lemierre syndrome, lemierre syndrome

## Abstract

Lemierre's syndrome is a rare but potentially severe complication of bacterial infections that usually affects previously healthy adolescents and young adults. It commonly presents as septic thrombophlebitis of the internal jugular vein and bacteremia following a recent oropharyngeal infection. The most commonly isolated organisms are *Fusobacterium necrophorum*, followed by *Fusobacterium nucleatum* and other anaerobes. Atypical Lemierre's syndrome is characterized by thrombophlebitis at sites distant from the head and neck veins and is far less encountered than typical Lemierre's syndrome. Here, we present a case of an elderly African American female with pylephlebitis, a rare abdominal variant of Lemierre's syndrome with extensive portal vein, splenic vein, and mesenteric vein thrombosis following perforated diverticulitis and resultant *F. nucleatum* bacteremia. She demonstrated complete recovery following appropriate long-term intravenous antibiotics and anticoagulation. This case calls attention to the re-emergence of the rare manifestation of this forgotten disease and highlights improved outcomes with prompt recognition and early treatment.

## Introduction

*Fusobacterium* species are gram-negative anaerobic bacilli that are commonly known for causing Lemierre's syndrome (LS). They frequently colonize the gastrointestinal tract, respiratory tract, and female genital tracts. These organisms, which are more virulent than most of the normal anaerobic flora, cause bacteremia and a variety of rapidly progressive infections.

One such famous association is LS, which is characterized by thrombophlebitis of the internal jugular vein caused primarily by *Fusobacterium necrophorum* and *Fusobacterium nucleatum. Fusobacterium *species are inherently thrombogenic in nature due to their ability to cause platelet aggregation through unclear mechanisms. Thrombus formation and rapid bacterial growth can cause septic embolization to distant sites. Mortality burden varied from 5% to 19% in patients afflicted with *Fusobacterium *species bacteremia. *F. necrophorum* was widely seen in younger individuals, and *F. nucleatum* was seen in older individuals with comorbidities and associated with gastrointestinal (GI) infections and/or malignancy.

Rarely seen is an abdominal variant of LS, with only a few reported cases existing in literature, which we have described below.

## Case presentation

**Figure 1:**
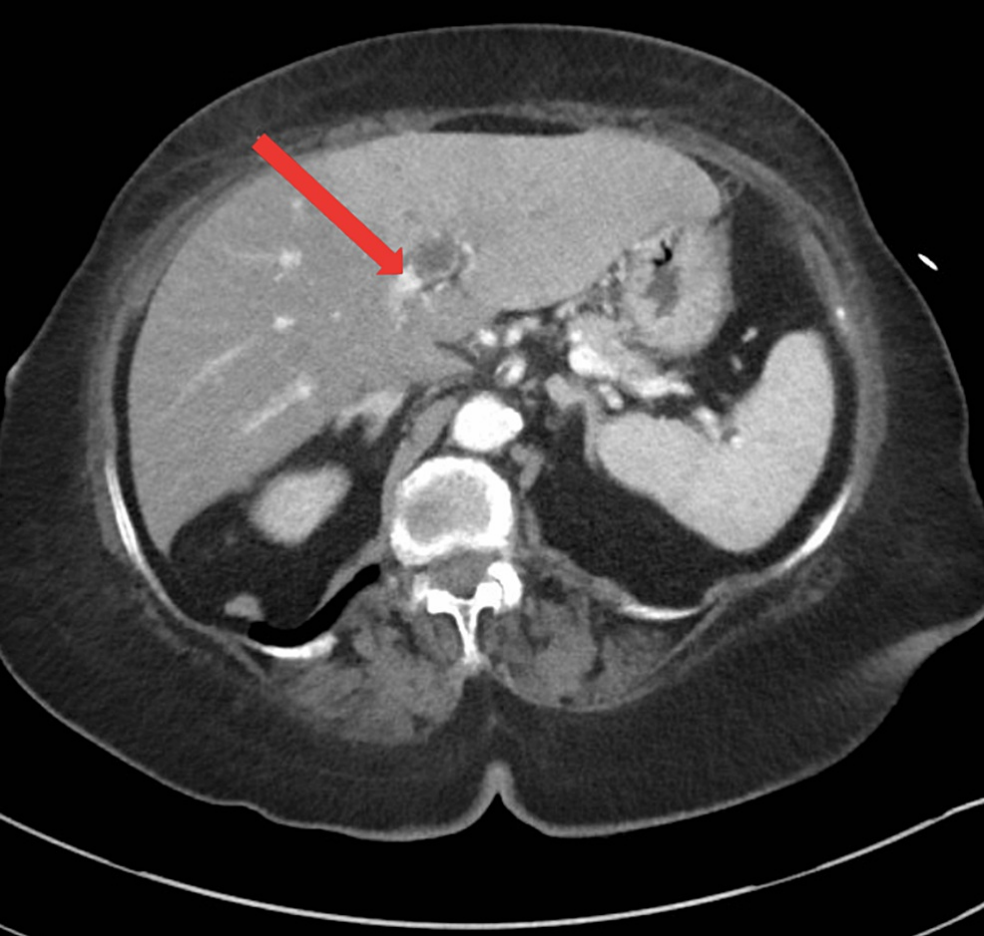
Initial computed tomography of the abdomen showing portal vein thrombosis

**Figure 2:**
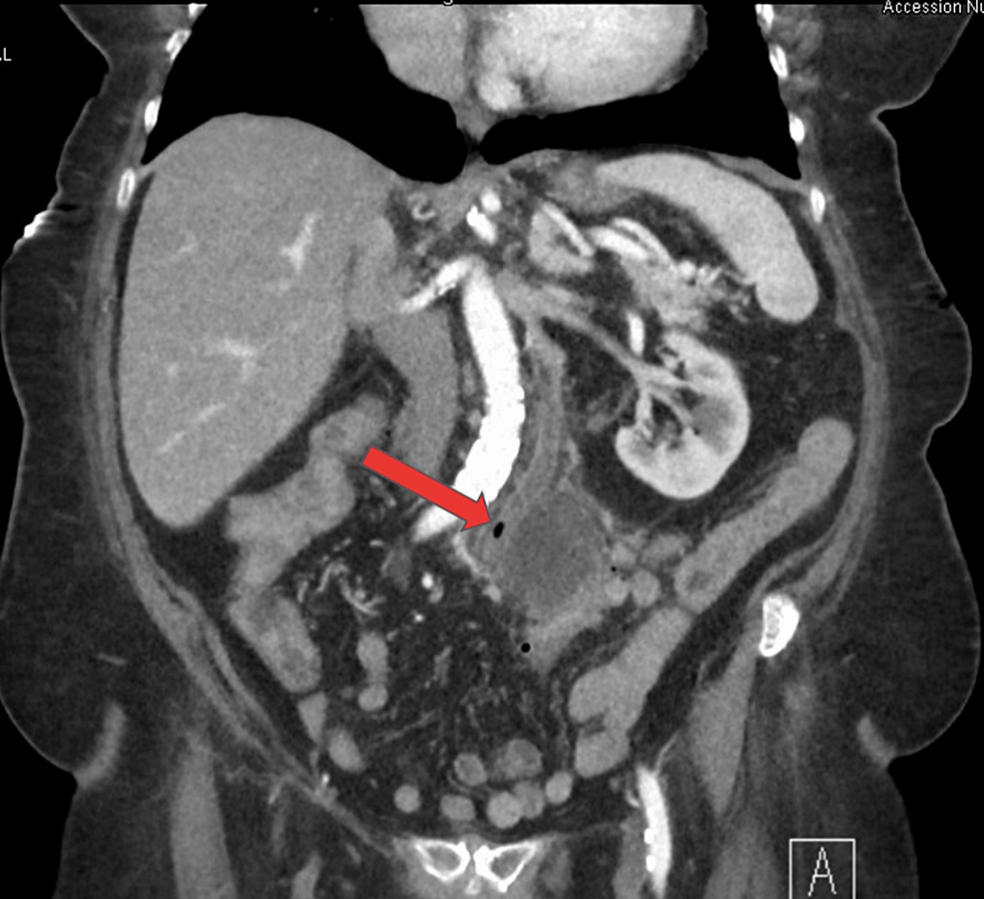
Initial computed tomography of the abdomen showing diverticular abscess and superior mesenteric vein thrombosis

An 86-year-old African American female with a past medical history of hypertension, dyslipidemia, diverticulosis, and recent severe lower GI bleeding presented to the emergency department with chief complaints of abdominal pain. The patient had a recent hospitalization three months prior, secondary to lower GI bleeding that required multiple blood transfusions. During the previous admission, she underwent colonoscopy, which revealed sigmoid diverticulosis with no signs of active bleeding. A red blood cell scan was negative as well. She was relatively fine till one week before her presentation when she started having progressive intermittent abdominal pain. She described the pain to be moderate in intensity, generalized but more prominent on the left lower quadrant, and not related to food consumption. She reported having associated subjective fever and chills and decreased appetite. A review of the system was negative for nausea, vomiting, melena, frank blood per rectum, diarrhea, or constipation. Physical examination was significant for normoactive bowel sound, left lower quadrant tenderness without guarding, or rebound tenderness. Initial assessment in the emergency room revealed a white blood cell (WBC) count of 16.2 K/uL with 93% polys, hemoglobin of 12 gm/dL, red cell distribution width (RDW) of 14.1%, lactic acid of 2 mmol/L, lipase at 35 U/L, alanine transaminase (ALT) at 60 U/L, aspartate aminotransferase (AST) at 78 U/L, alkaline phosphatase at 250 U/L, total bilirubin at 1.3 mg/dL, direct bilirubin at 0.7 mg/dL, albumin at 2.1 g/dL, creatinine at 1.77 mg/dL, blood urea nitrogen (BUN) at 42 mg/dL, and potassium level at 2.2 mmol/L. An electrocardiogram showed diffuse T wave inversion in anterior leads with a picture of severe hypokalemia, which improved the next day. Abdominal computed tomography (CT) scan was performed, which revealed a 4-cm abscess with an air-fluid level in an area of diverticular disease in the sigmoid colon, incomplete occlusive thrombus of the main portal vein with thrombosis of the left portal vein, splenic vein, and superior mesenteric vein (Figures 1, 2).

Blood cultures were sent, and the patient was started on antibiotic treatment with cefepime and metronidazole with renal dose adjustment. Heparin infusion was initiated for portal vein thrombosis as well. The patient was evaluated by the surgery team, who suggested that she is not a candidate for surgical intervention and recommended abscess drainage by interventional radiology (IR). Further evaluation was done and, unfortunately, the abscess was surrounded by bowel loops and was not accessible via a percutaneous approach. As a result, it was decided to manage the patient conservatively with antibiotics and clinical monitoring. The patient’s abdominal pain improved significantly with antibiotic treatment in a few days, and she could tolerate the diet. In view of previous severe GI bleeding and current heparin infusion, she was closely monitored for any signs of bleeding, which were not detected.

On day five, blood cultures that were obtained initially on admission were reported positive for *F. nucleatum*, which was sensitive to current antibiotic treatment. As *F. necrophorum* is associated with septic jugular vein phlebitis (Lemierre's syndrome), there were concerns that portal vein thrombosis could be secondary to current bacteremia. The patient was also found to have bilateral peroneal acute deep vein thrombosis (DVT) in the lower extremities venous duplex. She denied having any previous history of hyper-coagulopathies. Later, she was evaluated by a hematologist, who recommended outpatient follow-up for further hypercoagulable workup.

**Figure 3:**
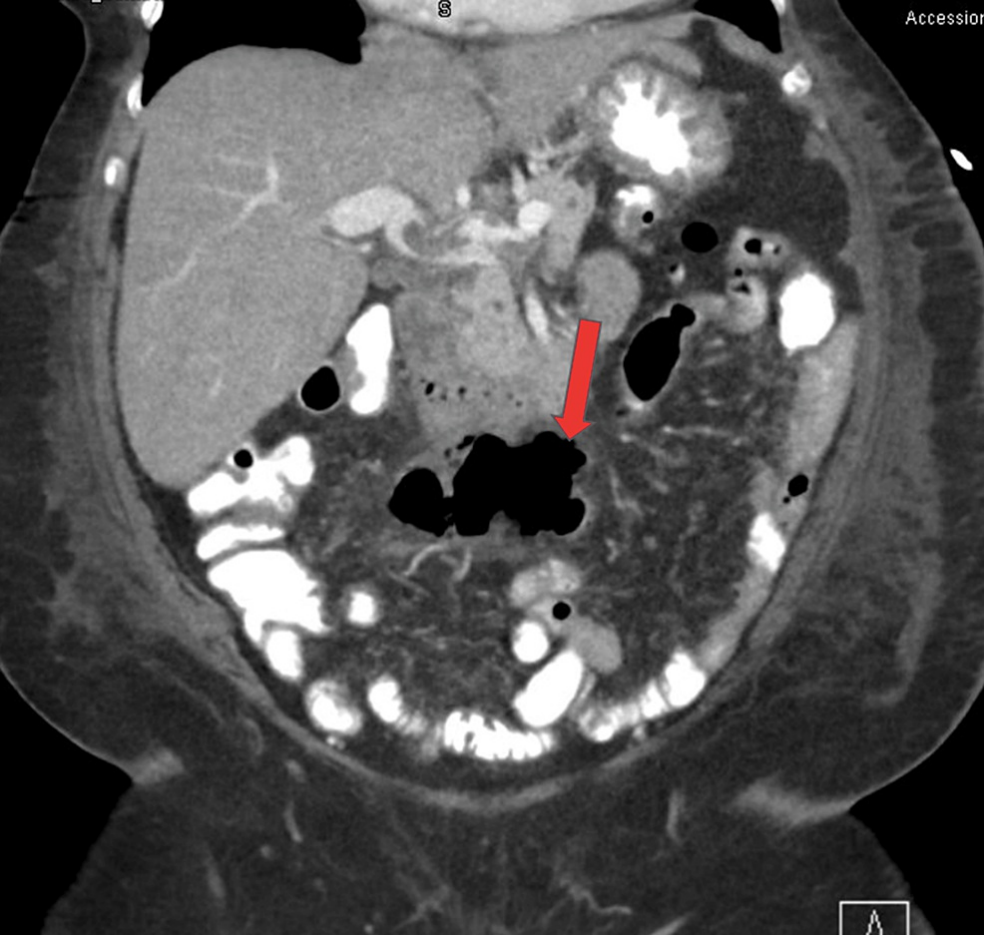
Second computed tomography of the abdomen showing enlarging mesenteric abscess extending from left to right lower quadrant

After one week of antibiotic treatment, the patient’s abdominal pain was completely resolved with no further tenderness on the examination. She remained afebrile during hospitalization and her WBC improved to 6.1 K/uL. CT of the abdomen was repeated on day seven to assess the resolution of the abscess and surprisingly showed enlarging mesenteric abscess measuring 7 x 3 cm extending from left to right lower quadrant, with no changes in the portal vein thrombosis (Figure 3).

The patient was re-evaluated by the surgery and IR team and was not a candidate for any intervention. Intravenous antibiotic treatment was continued for 13 days, and it was switched to oral amoxicillin-clavulanate for additional two weeks. The patient was started on oral apixaban for portal vein thrombosis and acute DVT. An abdominal CT scan was repeated on day 15, which was unchanged from the previous one. At the time of discharge, she was advised to follow up with her primary care physician and hematologist to repeat a CT scan of the abdomen and close monitoring.

## Discussion

*Fusobacterium* is an anaerobic, gram-negative bacterial genus that is commensal to the alimentary canal and genitourinary tract. As a pathogen, it is associated with several local and systemic complications. It is known to cause peritonsillar, diverticular, and liver abscesses. Its local invasion of the pharyngeal space and adjacent vasculature can cause septic thrombophlebitis of the internal jugular vein, which is often referred to as LS. The most common systemic complications are bacteremia and sepsis, but these microbes also have implications for Alzheimer’s disease, appendicitis, chorioamnionitis, colorectal cancer, inflammatory bowel disease, and thrombosis of various venous sites throughout the body [1].

In 2017, a study out of Spain described an annual incidence of *Fusobacterium *infection to be 1.78 per 100,000 and an annual incidence of *Fusobacterium* bacteremia to be 0.53 per 100,000. Studies from Canada, Finland, Sweden, and the United Kingdom all described a similar annual incidence of *Fusobacterium* bacteremia ranging from 0.50 to 0.76 per 100,000 [2-6]. Although *Fusobacterium* is most famously associated with its role in LS, this complication is seen in only about 0.05-0.09 cases per 100,000 annually [7]. Pylephlebitis, the abdominal variant of LS, is even rarer. From 1999 to 2019, there were only seven published case reports within the PubMed database describing septic portal vein thrombosis secondary to *Fusobacterium* species [8].

Pylephlebitis can result from any inflammatory process within the abdomen, but pancreatitis and diverticulitis are the most common causes. One retrospective study from the Mayo Clinic described 95 patients with portal vein thrombosis within a 10-year period. Bacteremia was present in 44% of those cases with *Streptococcus viridans*, *Escherichia coli*, and *Bacteroides fragilis* being the most common culprits. *Fusobacterium* species were isolated in less than 5% of those blood samples. Some studies suggest a tendency for *F. necrophorum* to affect mostly younger individuals with no past medical history. Those same reports describe *F. nucleatum *bacteremia to be associated with older persons who have underlying comorbidities like gastrointestinal malignancies and renal failure [4,9]. However, there are conflicting data on these associations [8,10].

Pylephlebitis is managed with antibiotics and anticoagulation. Empiric treatment should cover anaerobes and can include a third-generation cephalosporin plus metronidazole. Isolated *Fusobacterium* species can be treated with a carbapenem or beta-lactam/beta-lactamase inhibitor combination like piperacillin-tazobactam. Four to six weeks of intravenous administration is recommended. In the literature review, there are controversies surrounding the role of anticoagulation in pylephlebitis. Anticoagulation is generally recommended due to the risk of mesenteric ischemia, but bleeding risk must be weighed in the setting of cirrhosis or known chronic portal vein thrombosis. Currently, there is no definite guideline for anticoagulation. However, patients with pylephlebitis and hypercoagulable state, and those with normal clothing factors and mesenteric vein thrombosis, will benefit from anticoagulation. In the setting of isolated portal vein thrombosis and normal clotting function, anticoagulation may be unnecessary. The mortality rate from septic pylephlebitis has improved over the past three decades. In 1995, the overall mortality of 19 patients with pylephlebitis resulting from various bacterial species was 32% [11]. The mortality rate in patients with pylephlebitis resulting from polymicrobial infection improved to 19% in 2010 and 11% in 2016 [12,13]. The improvement in mortality rate could be due to advances in diagnostic imaging and subsequent temporal improvement in antibiotic and anticoagulation administration. A comparison of presentation, management, and outcome of similar cases in the literature is provided in Table 1 [8].

**Table 1:** Summary of the presentation, treatment, and outcome of the seven published case reports within the PubMed database from 1999 to 2019 describing septic portal vein thrombosis secondary to Fusobacterium species Adapted from [8]. AST: aspartate aminotransferase; ALT: alanine transaminase; ALP: alkaline phosphatase; Cipro: ciprofloxacin; Clinda: clindamycin; Metro: metronidazole; PCN: penicillin; Pip: piperacillin; NA: not available; SMV: superior mesenteric vein; SV: splenic vein; Tazo: tazobactam; THET: transhepatic endovascular thrombolysis; Vanc: vancomycin; WNL: within normal limits.

Study	Age	Presentation	AST	ALT	ALP	Primary site of infection	Positive blood culture	Intra-abdominal aspiration culture	Additional thrombosis site	Antibiotic and duration (weeks)	Anticoagulant and duration (months)	Outcome
Soo (1999)	31	Fever, abdominal pain, leukocytosis	79	133	295	GI tract	Yes	No	SMV	Cipro + Metro (1), Augmentin (6)	Warfarin (6)	Full recovery
Clark (2003)	19	Fever, abdominal pain, leukocytosis	NA	52	331	Gynecological procedure or pharynx	Yes	Yes	SMV	Cipro + Metro + PCN (6.5)	Warfarin (long term)	Residual portal hypertension
Redford (2005)	53	Fever, abdominal pain, leukocytosis, alcoholism	75	38	194	Unknown	Yes	No	No	Metro + PCN (2), Clinda (5)	Warfarin (3)	Full recovery
Shahani (2011)	34	Abdominal pain, leukocytosis, alcoholism	WNL	WNL	WNL	Pancreas	No	Yes	SMV, SV	Vanc + Metro + tigecycline (4)	None	Improved
DePetrillo (2014)	53	Fever, abdominal pain, leukocytosis, alcoholism	NA	70	152	Unknown	Yes	No	No	Ertapenem (4)	Warfarin	Improved
Akhrass (2015)	32	Fever, abdominal pain, leukocytosis	WNL	WNL	WNL	Appendix	Yes	No	SMV, SV	Vanc, Pip/Tazo, Metro, Clinda	THET, warfarin (6)	Full recovery
Radovanovic (2018)	69	Fever, abdominal pain, leukocytosis, alcoholism	39	68	518	Oral cavity	Yes	Yes	Hepatic vein	Ceftriaxone + Metro (3), Augmentin (2)	Warfarin (3)	Improved

## Conclusions

Despite being rare, *F. nucleatum *bacteremia can be associated with thrombosis in atypical locations. In this article, we are aiming to increase physicians' awareness of a rare presentation of the abdominal variant of LS that requires prompt antibiotic and anticoagulation treatment for a favorable result.
